# Accelerating Health Data Sharing: A Solution Based on the Internet of Things and Distributed Ledger Technologies

**DOI:** 10.2196/13583

**Published:** 2019-06-06

**Authors:** Xiaochen Zheng, Shengjing Sun, Raghava Rao Mukkamala, Ravi Vatrapu, Joaquín Ordieres-Meré

**Affiliations:** 1 Escuela Técnica Superior de Ingenieros Industriales Universidad Politécnica de Madrid Madrid Spain; 2 Centre for Business Data Analytics Department of Digitalization Copenhagen Business School Copenhagen Denmark; 3 Department of Technology Kristiania University College Oslo Norway

**Keywords:** Internet of Things, distributed ledger technologies, data sharing, health information interoperability, IOTA Tangle, masked authenticated messaging, blockchain, intelligent healthcare

## Abstract

**Background:**

Huge amounts of health-related data are generated every moment with the rapid development of Internet of Things (IoT) and wearable technologies. These big health data contain great value and can bring benefit to all stakeholders in the health care ecosystem. Currently, most of these data are siloed and fragmented in different health care systems or public and private databases. It prevents the fulfillment of intelligent health care inspired by these big data. Security and privacy concerns and the lack of ensured authenticity trails of data bring even more obstacles to health data sharing. With a decentralized and consensus-driven nature, distributed ledger technologies (DLTs) provide reliable solutions such as blockchain, Ethereum, and IOTA Tangle to facilitate the health care data sharing.

**Objective:**

This study aimed to develop a health-related data sharing system by integrating IoT and DLT to enable secure, fee-less, tamper-resistant, highly-scalable, and granularly-controllable health data exchange, as well as build a prototype and conduct experiments to verify the feasibility of the proposed solution.

**Methods:**

The health-related data are generated by 2 types of IoT devices: wearable devices and stationary air quality sensors. The data sharing mechanism is enabled by IOTA’s distributed ledger, the Tangle, which is a directed acyclic graph. Masked Authenticated Messaging (MAM) is adopted to facilitate data communications among different parties. Merkle Hash Tree is used for data encryption and verification.

**Results:**

A prototype system was built according to the proposed solution. It uses a smartwatch and multiple air sensors as the sensing layer; a smartphone and a single-board computer (Raspberry Pi) as the gateway; and a local server for data publishing. The prototype was applied to the remote diagnosis of tremor disease. The results proved that the solution could enable costless data integrity and flexible access management during data sharing.

**Conclusions:**

DLT integrated with IoT technologies could greatly improve the health-related data sharing. The proposed solution based on IOTA Tangle and MAM could overcome many challenges faced by other traditional blockchain-based solutions in terms of cost, efficiency, scalability, and flexibility in data access management. This study also showed the possibility of fully decentralized health data sharing by replacing the local server with edge computing devices.

## Introduction

### Internet of Things and Intelligent Health Care

Internet of Things (IoT) has been developing explosively in recent years. It is believed to be the next revolutionary technology and bring great benefits to various domains of the society including health care [1]. The health care industry has been dramatically changed because of the information technology revolution that started in the last century. New technologies such as telemedicine, digital hospital, electronic health and mobile health have been widely applied during the past decades, and now, the rapidly development of IoT is promoting health care from digital into intelligent [2].

The advances of IoT have resulted in rapid emergence of smart environments such as smart home [3]. Sensors in these environments can measure the values of various environmental factors including temperature, humidity, air quality, and noise [4].

As an important aspect of IoT, wearable technology has also shown a surge in the past decade. Different types of wearable devices containing various embedded sensors such as smartphone, smart watch, smart band, and smart glasses have been used in health care applications to realize various health-related applications such as remote diagnosis [5], disease monitoring [6], and elderly people caring [7].

### Challenges of Health Care Data Sharing

Large amounts of health-related data are generated by these smart devices including environmental data from stationary sensors and activity data from wearable devices. These data are valuable resources for health care applications, research, and commercial projects. Properly sharing these health data can benefit all related stakeholders including the device users, patients, researchers, and companies and improve the public health care system.

Currently, most data generated by IoT devices are controlled by different service providers, device manufacturers, or scattered in different health care systems [8,9]. These siloed and segmented data make it impossible or very difficult to share data outside their own closed environments, and this leads to enormous quantities of wasted data [10]. Besides, it puts data security and privacy at risk as these centralized data stores and authority providers are attractive targets for cyberattacks [11].

With the increasing concern about data privacy and security issue from public and private users, data protection regulations will become stricter. For example, the European Union has published the General Data Protection Regulation [12] to protect individual data. Such regulations make data sharing even more difficult.

Besides the complex data protection regulations, another main obstacle to freely flowing of big data is that, although data sharing is becoming cheaper from a technological perspective, it is prohibitively expensive to transfer fine, granular data in real time because of intermediary fees [13]. Another barrier is the lack of ensured authenticity and audit trails of data. Traditional data transmission protocols and databases are susceptible to various attacks, including *man-in-the-middle* attacks and data tampering [14].

To overcome these barriers that hinder the full use of valuable health data, it is necessary to develop advanced systems to accelerate secure, fee-less, tamper-resist, and high-scalable health data sharing.

### Distributed Ledger Technologies and Blockchain

A distributed ledger is a distributed database, maintained by a consensus protocol run by nodes in a peer-to-peer network. This consensus protocol replaces a central administrator, as all peers contribute to maintaining the integrity of the database [15].

As one of the most widespread DLT, the blockchain, has gained substantial popularity in recent years, primarily in the financial field because of the cryptocurrencies. For example, Bitcoin was first introduced in 2008 [16] and ever since has attracted the attention of the research community from diverse academic fields [17-19] and gained mainstream popularity because of its unique characteristics such as the absence of centralized control, an assumed high degree of anonymity, and distributed consensus over decentralized networks. Blockchain solutions could reduce data breach risks by utilizing threshold encryption of data together using public key infrastructure, where cooperation of multiple parties is required to decrypt data, and asymmetric cryptography is used to authenticate communication with system participants [20].

### Limitations of Blockchain

Specialized distributed consensus protocols based on DLT have enabled novel decentralized applications such as cryptographic currencies [16] and smart contracts [21]. The rise and success of Bitcoin during the last 6 years proved that blockchain technology has real-world value. However, these block-based protocols, such as blockchain and Ethereum, also have several drawbacks that prevent them from being used as a generic platform for IoT data sharing.

#### Scalability

A blockchain has an inherent transaction rate limit because all participants agree on the longest chain and discard forks and side branches [22]. Common practice is to wait for 6 blocks to be added to the longest chain before reaching a high level of confidence that a transaction is final on the Bitcoin network [20,23]. As an example, it took on average 9.3 min to confirm a Bitcoin transaction at the end of December 2018 [24]. Applications that require exchange of value and low latency cannot be certain that their transactions are final in a shorter time frame and must trust the payer to not double spend [20]. The current incentive schemes that allow these protocols to spread virally make inefficient use of computational resources while constraining the transaction rate on the network. The transaction rate of Bitcoin protocol has been lower than 5 transactions per second in the whole network during most of the time in the year 2018 [25]. Similarly, the Ethereum protocol currently processes about 6 transactions per second across the entire network [26]. This low throughput cannot fill the requirements of data sharing in many health care scenarios.

#### Fees

Another notable drawback is the concept of a transaction fee for transactions of any value. For example, the Bitcoin protocol requires a fee that may exceed US $0.30 each transaction [27] according to the statistics of January 10, 2019. To use a distributed ledger at scale for financial or other industrial use cases, this low throughput and high fee model will not suffice. The importance of micropayments will increase in the rapidly developing IoT technology and paying a fee that is larger than the amount of value being transferred is not logical. Furthermore, it is not easy to get rid of fees in the blockchain infrastructure as they serve as an incentive for the creators of blocks [28].

#### Centralization

Lots of computing power is required to maintain the blockchain, and mining power has become centralized to some extent. The latest statistic shows that the 6 largest mining pools control 75.76% of the of the network’s mining power (BTC.com 21.5%, AntPool 14.9%, SlushPool 11.01%, ViaBTC 10.65%, BTC.TOP 9.67%, F2Pool 8.03%) [29].

#### Vulnerable to Quantum Attack

Bitcoin and other proof-of-work–based blockchains are susceptible to being broken by quantum computers. Quantum computers, although still a hypothetical construct as of today, could be very efficient for handling problems that rely on trial and error to find a solution [28]. The process of finding a nonce to generate a Bitcoin block is a good example of such a problem. As of today, one must check an average of 2^68^ nonce to find a suitable hash that allows a new block to be generated. Theoretically, a quantum computer would need θ(√N) operations to solve a problem that is analogous to the Bitcoin puzzle stated above [30]. This same problem would need θ(√N) operations on a classical computer. Therefore, a quantum computer would be around 17 billion (√2^68^) times more efficient at mining the Bitcoin blockchain than a classical computer. It would make possible of gaining control of over 51% of computing power of the whole blockchain network, which would enable attackers to double spend and break the entire network.

### IOTA and the Tangle

IOTA is a tangle-based cryptocurrency designed specifically for the IoT industry where a machine-to-machine micropayment system is required. The tangle naturally succeeds the blockchain as its next evolutionary step by overcoming some of its previously mentioned fundamental limitations [31]. The main feature of the tangle is that it uses a directed acyclic graph for storing transactions instead of sequential blocks. In the Tangle, users must perform a small amount of computational work to approve 2 previous transactions to issue a new transaction. This new transaction will be validated by some subsequent transactions [28].

This structure enables the Tangle with high scalability. There is no maximum throughput, as the more activities in the Tangle, the faster transactions can be confirmed. In addition, with this 'pay-it-forward' system of validations, there is no need to offer financial rewards. Transacting with IOTA can be free of charge. Moreover, IOTA has no miners, therefore it is truly decentralized.

The IOTA tangle is designed to be quantum resistant. The number of nonce that one needs to check to find a suitable hash for issuing a transaction is around 3^8^ on average, which is not unreasonably large. The gain of efficiency for an “ideal” quantum computer would therefore be of order 3^4^=81, which is already quite acceptable [28]. More importantly, the algorithm used in the IOTA implementation is structured such that the time to find a nonce is not much longer than the time needed for other tasks that are necessary to issue a transaction. The latter part is much more resistant against quantum computing compared with the traditional blockchain.

### Masked Authenticated Messaging

The main data communication protocol used in the proposed system is Masked Authenticated Messaging (MAM). It enables to emit and access an encrypted data stream over the Tangle regardless of the size or cost of a device [32]. MAM uses channels for message spreading. IOTA users can create a channel and publish a message of any size at any time. A small amount of proof-of-work is required to allow the data to propagate through the network and to prevent spamming. Other users can subscribe this channel through its address and receive a message that is published by the channel owner.

#### Merkle Hash Tree

MAM uses a signature scheme based on Merkle Hash Tree (MHT) [33-35] to sign the cipher digest of an encrypted message [32]. The *address* of a channel is the *root* of this Merkle tree, which itself is created using the *seed* of the user.

**Figure 1:**
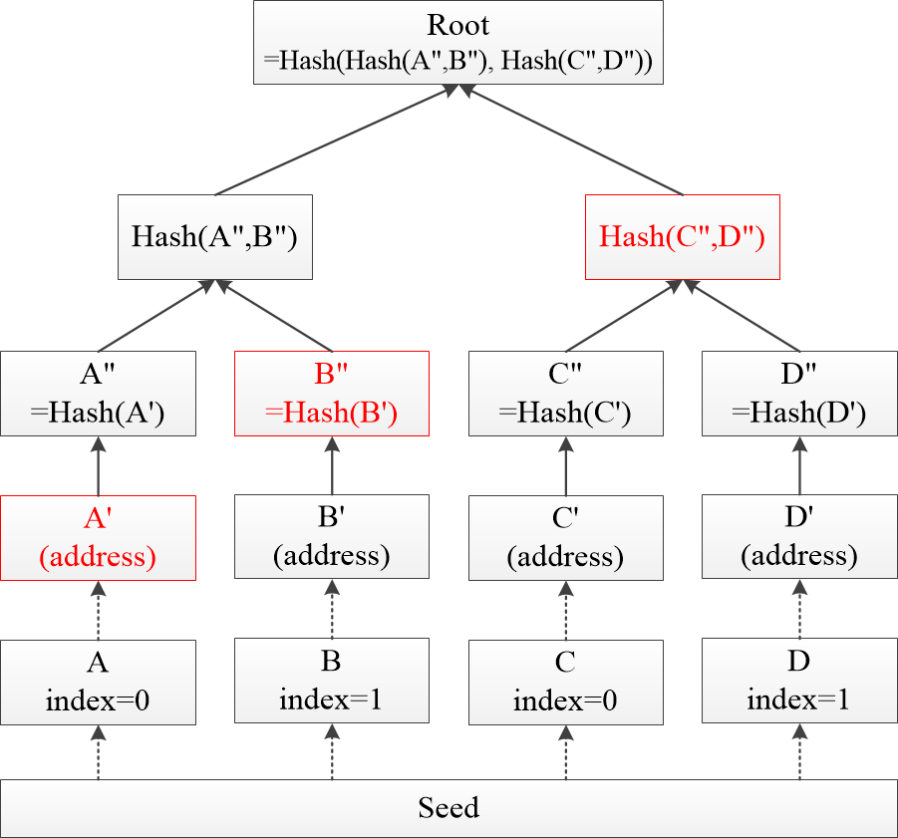
An example of Merkle Hash Tree with 4 leaves.

As the MHT example shown in Figure 1, private keys (A, B, C, D) are generated according to the *seed*, *index*, and *security level* [36]. The corresponding *addresses,* also called *leaves* (A', B', C', D'), can be generated respectively [37,38]. By applying the hash functions to narrow the addresses, the *root* of the Merkle tree can be obtained. In a MAM stream, a single MHT only lasts for a short period of time, each message contains the root of the next Merkle tree (or the future direction of the channel) [32].

Each message is signed with the one-time signature (OTS) scheme. Each leaf in the MHT corresponds to 1 OTS scheme. This means that each tree can produce the same number of messages as the number of leaves in the MHT [15].

In an MHT, the set of complementary hashes of a given leaf are the *siblings* of this leaf. As shown in Figure 1, the siblings of leaf *A'* (in red color) are *B“* and *Hash(C”,D“)*. By combining a given leaf and its siblings, the *root* of an MHT can be calculated.

**Figure 2:**
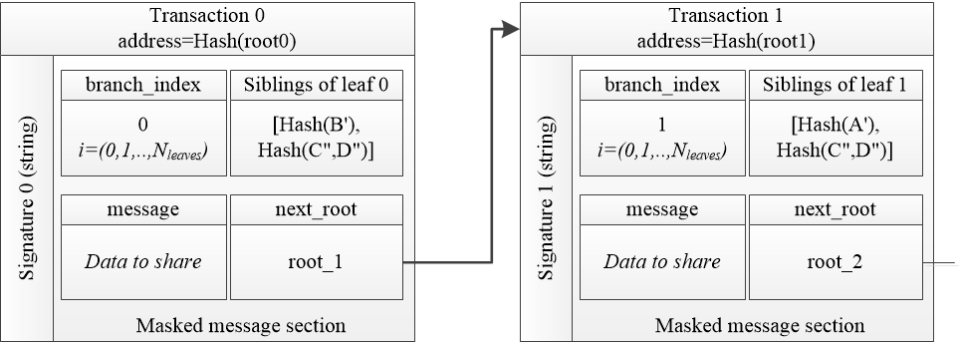
Structure of a Masked Authenticated Messaging stream with 2 transactions.

A complete MAM transaction should include a signature section and the masked message section. The signature is created from one of the private keys corresponding to one of the leaves. The masked message consists of the raw data that need to be shared, the root of the next MHT, the index of the chosen leaf (branch index), and the siblings of this leaf. Figure 2 shows an example of a MAM stream with 2 transactions.

#### Privacy and Encryption Modes

MAM has 3 privacy and encryption modes to control the visibility and access of a channel: public, restricted, and private.

In public mode, the root of MHT is directly used as MAM transaction address and channel key. Therefore, any user who receives a message randomly or intentionally can then decode it by using the address of the message.

In private mode, the hash of the MHT root is used as the address, and the message is decrypted using the *root*. This prevents random users from decrypting the message if they stumble across it as they are unable to derive the *root* from the hash.

In restricted mode, an authorization key, named as *sideKey* in this study, is added based on private mode. The address used to attach to the network is the hash of the *sideKey* and the *root* (according to the current MAM source code [38], only the hash of root is used, which differs from the introduction of IOTA website [32,36]). It enables a message publisher to revoke access to future messages from subscribers by changing the *sideKey*.

To consume a MAM message, the receiver needs to use the *root* to calculate the address of the transaction and fetch the masked message. Then, use the *root*, and *sideKey* in restricted mode, to decrypt the masked message.

In a MAM channel, the current message contains the address of the following message, whereas the previous ones are not referenced. This adds the forward secrecy character to a channel. When users are authorized with the correct decryption key, they could follow a MAM stream from the current transaction, but there is no way to read previous messages.

### Objective of This Paper

The objective of this study was to integrate IOTA Tangle with IoT to develop a health data sharing system, which could support secure, fee-less, tamper-resist, high-scalable, and granular-controllable health data exchange. The data source could include both wearable devices and stationary sensors in a smart environment such as smart home. The feasibility of the proposed system needs to be verified with a prototype system and its application in a practical case.

## Methods

### System Architecture

**Figure 3:**
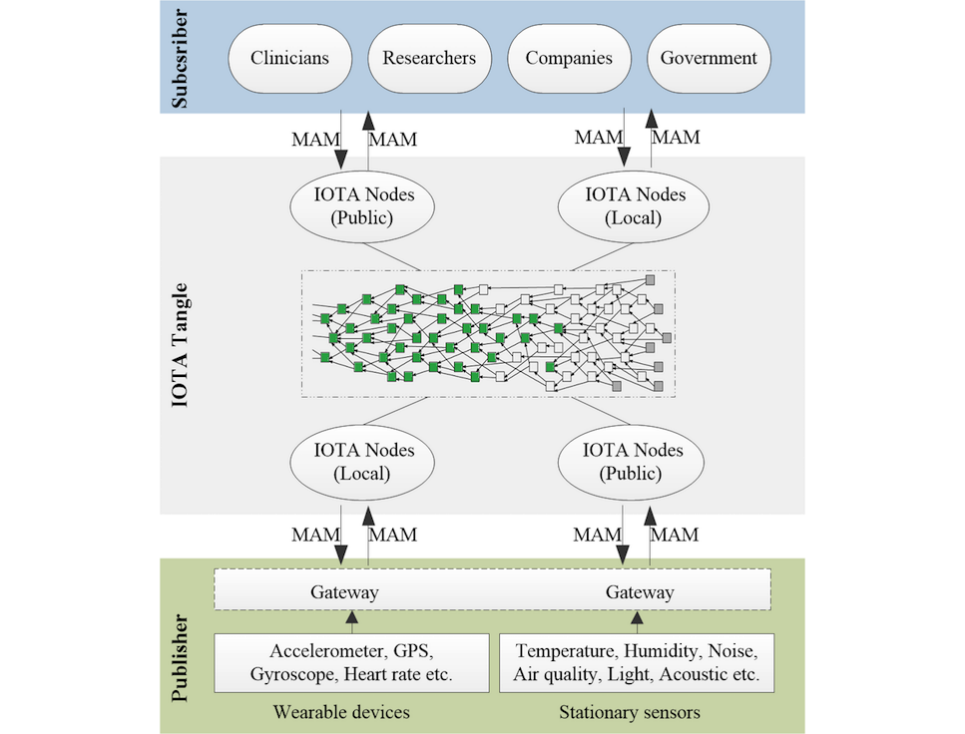
Architecture of the proposed health data sharing system based on IOTA Tangle. GPS: Global Positioning System; MAM: Masked Authenticated Messaging.

The architecture of the proposed health care data sharing system is presented in Figure 3. There are 2 roles involved in this system, data publisher and data subscriber. The publisher can be an individual, a family, or any other organization who possesses smart devices and sensors. These devices, sensors, and their owners produce health-related data, which are then published to the Tangle using specific encryption and privacy protocols. The data are published in their own channels, and each channel has an address. The subscribers of a data channel will receive the new published data. The published data are usually encrypted, and an extra decryption key may be necessary to decrypt the received data.

All the data are published and received through an IOTA node, which is a computer connected to the IOTA network. Users may use their own node or use public nodes. A user can be a data publisher and a subscriber at the same time. For example, a patient can publish his or her health data, and his or her doctor can subscribe these data and make evaluation accordingly. Afterwards, the doctor can publish the evaluation result to the Tangle, and the patient can subscribe this channel and receive the result.

Due to the limitations of size, power supply, and computing capability, most wearable devices and environmental sensors cannot publish or receive data directly to or from the Tangle. In this case, a gateway layer will be necessary, which could be a computer, a smartphone, or a single-board computer such as Raspberry Pi [39].

### Implementation

**Figure 4:**
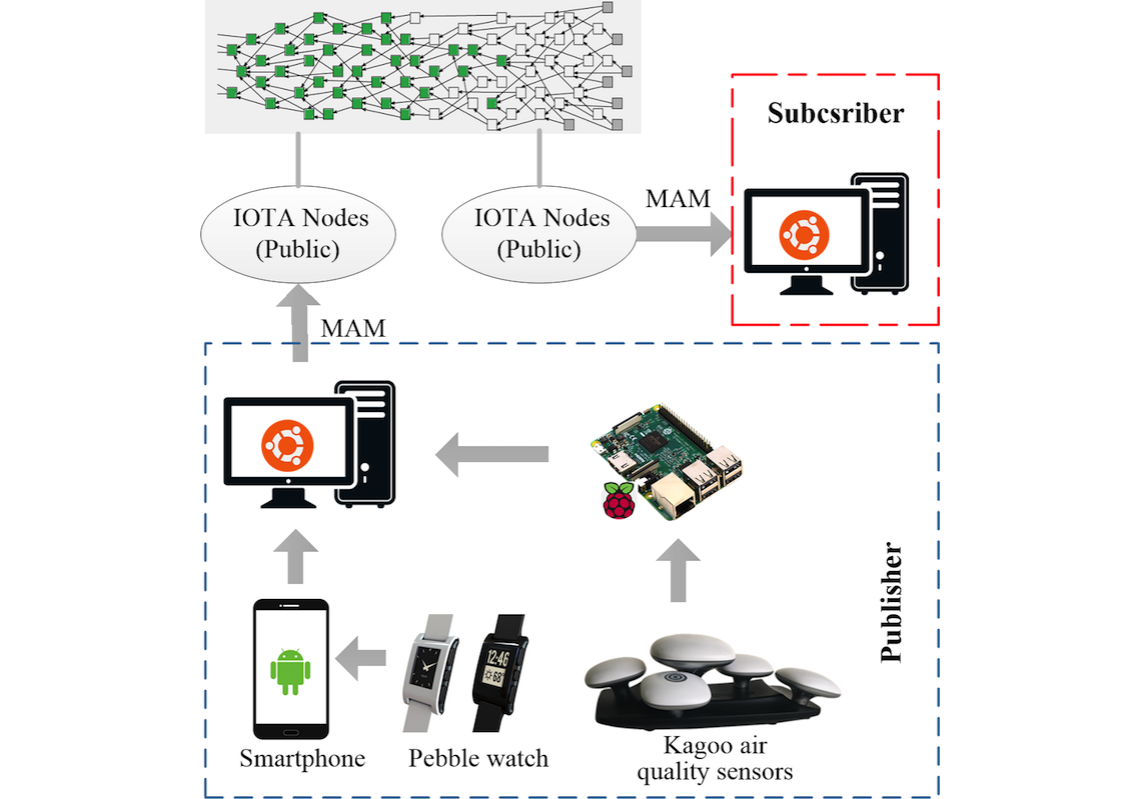
Prototype of the health data sharing system using smartwatches, smartphone, air sensors, and Raspberry Pi. MAM: Masked Authenticated Messaging.

To verify the feasibility of the proposed health data sharing system and demonstrate the implementation process, a prototype has been developed. The structure of the prototype is shown in Figure 4.

A portable human movement monitoring system using smartwatches was developed previously for the remote diagnosis of essential tremor (ET) [40,41]. The Pebble smartwatch [42] in this system could measure the triaxial acceleration data for tremor evaluation and activity recognition. The customized apps in the smartphone allows users to report their location, activity name, tremor level, self-evaluation about the disease, and other factors related to the disease, such as medication, alcohol, and coffee intake. These data, after integrated with other sensor data generated by the smartphone, will be compressed and uploaded to the remote server via internet for analysis using machine learning techniques.

In addition to the movement monitoring system, we added an environmental monitoring system composed of Kagoo air quality sensors [43] and Raspberry Pi [39]. The Kagoo sensors could measure various environmental factors such as temperature, humidity, noise, and the content of pollutions in the air, including particulate matter, formaldehyde, total volatile organic components, benzene, carbon dioxide, carbon monoxide, ozone, and nitrogen dioxide. These sensors can be freely combined and plugged into a motherboard, which can communicate with the single-board computer Raspberry Pi through wired or Wi-Fi connection. A Python program running on Raspberry Pi could fetch and preprocess the environmental data from air sensors. More technical details, including the hardware manual and software codes, are openly accessible [44].

We use these 2 data collecting systems to represent wearable devices and stationary context sensors. The combination of these 2 data sources could provide a more complete understanding about users’ health-related information.

In this prototype, the Pebble smartwatch, Android smartphone, and air quality sensors compose the sensing layer; the smartphone and Raspberry Pi play the role of gateway corresponding to the architecture of the proposed system. The data collecting frequency varies among different devices. The acceleration data from the smartwatch are recorded with a frequency of 25 Hz and uploaded every minute in a batch. The frequency of the data from smartphone depends on user’s habit and usually is less than once per hour. The Kagoo sensors record environmental data once per minute.

In this prototype, instead of directly published to the Tangle through the gateway as shown in Figure 1, the raw data are first sent to a local server for processing. The reasons are 2-fold. First, the frequency of the acceleration data from smartwatch is much higher than the other 2 data sources. Publishing these raw data to the Tangle will lead to a long lagging period. Therefore, on the server side, the acceleration data will pass through a tremor evaluation module based on deep learning approaches [41]. The output will be a shorter message per minute with a time stamp and a tremor score based on the classification result. This shorter message will be published to the Tangle. The raw acceleration data will be saved in a private database for future use. The second reason is to simplify the experiment of testing the average waiting time of publishing messages. The data from different sources are all gathered in the server and published through the same node in a concentrated period to obtain a more reliable result.

In terms of privacy and encryption modes, the environmental data are published in public MAM mode, whereas the patient report data and tremor evaluation data are published in restricted MAM mode.

To consume the published data over the Tangle, subscribers only need to know the address of the channel if the data are published in public mode, whereas an extra decryption key is needed for the data in restricted mode as introduced previously. Both data publishing and data receiving were realized through the JavaScript programs, which are introduced with details in the following experiment and results section.

## Results

### Experiment

An experiment was conducted to prove the feasibility of the proposed system, which can broadcast and receive combined health data from both wearable devices and stationary environmental sensors. In general, 3 types of data are tested, including tremor level based on smartwatch acceleration data, patient reports from smartphone, and environmental data from air sensors. The environmental data were broadcasted using public MAM protocol, whereas the other 2 types of data were broadcasted using restricted MAM protocol. The authentication keys of restricted mode were changed during a broadcast stream to demonstrate how a user could revoke access to the data they generate in future.

All the data were broadcasted and received in JSON format. For each type of data, 100 trials of broadcasting were realized to test the average waiting time. The data were published using a computer equipped with a 4-core Intel Core i5-4460 3.2 GHz CPU, a 12 GB of RAM memory, and the Ubuntu Linux 18 64-bit version operating system. The data were published through a public IOTA node [45,46]. The memory usage was 50%, and the number of neighbors was 12 when connected to this node during the experiment. The complete scripts for publishing and receiving JSON data over the Tangle are openly available [47].

### Experiment Outcomes

**Figure 5:**
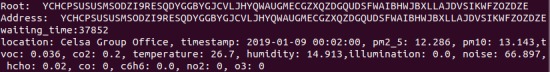
Environmental data published to the Tangle with public Masked Authenticated Messaging mode.

Figure 5 shows an example of published environmental data over the Tangle using public MAM mode. It displays that the *address* of the channel is the same as the *root* of the MHT. Any user who knows the *address* could fetch the message and decrypt it with *root*, which is the same to the *address*.

**Figure 6:**
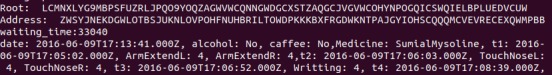
Patient report data published to the Tangle with restricted Masked Authenticated Messaging mode.

Figure 6 presents an example of patient report data published in restricted mode. In this case, the *address* is the hash of the MHT *root*, which is totally different. Subscribers need to know both the *address* and the extra encryption key (*side_key*) to fetch and decrypt the message. In restricted mode, the publisher can send a subscriber the *address* and *side_key* to grant him or her access to the current and future messages in the data stream. To revoke the authorization, the publisher just needs to change the *side_key* when publishing a new message, and subscribers without the new *side_key* will loss the access to this message and future ones.

**Figure 7:**
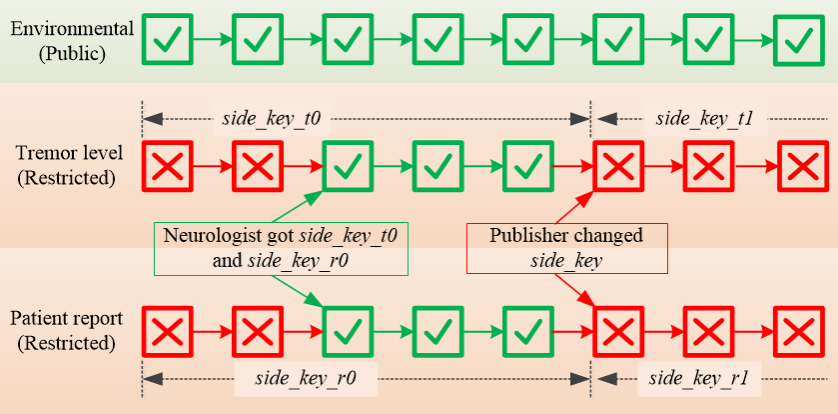
Granular access control over messages published over the Tangle by combing public and restricted Masked Authenticated Messaging protocol.

The combination of public and restricted MAM protocols could provide users granular control over their heath data, which could bring great benefit to the health care system. For instance, in our prototype, when a patient wants to be diagnosed, he or she can share with the neurologist the *address* and the *side_key* to the report data and tremor evaluation data streams from a certain time. Afterwards, the neurologist will be able to fetch all 3 data streams as the environmental data are published in public mode. After the diagnosis, the patient can change the key to revoke the authorization, as shown in Figure 7.

**Table 1:** Result of the data broadcasting experiment using Masked Authenticated Messaging (MAM) protocol.

Data	MAM mode	Size (bytes)	Waiting time per message (seconds)		
			Mean (SD)	Maximum	Minimum
Air quality	Public	260	20.41 (8.11)	55.41	8.81
Tremor level	Restricted	29	17.35 (5.37)	33.41	7.81
Personal report	Restricted	570	19.99 (7.61)	55.17	10.10
Air quality from Raspberry Pi	Public	260	22.56 (5.97)	40.36	12.87

The summary of the waiting time for publishing the 3 types of data to the Tangle based on 100 trials is presented in Table 1. The result shows that there is no obvious difference among these 3 types of data in terms of waiting time for publishing to the Tangle, although their message length and encryption modes are different. This is because of the fact that, in IOTA Tangle, the size of a transaction is 2673 trytes, which is about 1650 bytes. It means that as far as a message is shorter than this limit, the waiting time of publishing such messages should be similar on the same node. The actual waiting time depends on the computing capability of the node to perform proof of work, and it may vary from a few seconds to more than half minute according to our tests as shown in Table 1.

Currently, the bottleneck of the data publishing speed is the total number of nodes connected to the Tangle network and the condition of the specific node used by the publishing device. It is expected that the time to publish data from a local server or from the single board computer should be similar. Aiming to verify this consumption, an extra test using Raspberry Pi to publish the air quality data was conducted in addition to the experiment of publishing 3 types of data using a local server. The result is presented in Table 1. It shows that there is no obvious difference regarding the waiting time for publishing a message, which verified the aforementioned consumption.

## Discussion

### Principal Findings

This study explored the application of emerging distributed ledger technology in the health care domain. We proposed a health data sharing system by converging IoT, IOTA Tangle, and MAM protocol. It makes possible of a reliable marketplace for the individuals to share their health-related data with hospitals, researcher, industry companies, or any other organizations in a secure and controllable way. In return, individuals can get benefit from their own data in monetary, medical services, or other forms. On the other hand, researchers and companies will be able to gather relevant data for their studies, clinical trials, or product development.

Most existing studies about the applications of DLT and IOT in health care either focused on the conceptual design of health data sharing systems or discussed relevant policies from managerial perspectives. In comparison, this study not only proposed an application framework supported by DLT and IOT technologies but also implemented a prototype system in practice from technical perspective.

Through an experiment based on a prototype system, we demonstrated how the health-related data are collected and published to the Tangle in different encryption and privacy options. Our experiment showed that combining public and restricted MAM data streams, individuals are enabled to define granular access controls to different data consumers. The proposed system could facilitate the development of fee-less, secure, and efficient health data sharing marketplace to handle the big data generated by numerous IoT devices, and hence, pave way to the promising intelligent health care.

Although the current implementation of IOTA Tangle and its MAM protocol are already usable, they are still under development and are evolving rapidly. The current waiting time for attaching a message to the Tangle may vary from a few seconds up to more than 1 min. Although this is faster than other block-based protocols, there is still large room for performance improvements, as the more nodes connected to the Tangle network, the faster a transaction can be approved.

### Limitations

The feasibility of the proposed health data sharing system using IOTA Tangle and MAM protocol was verified through the experiments based on a prototype system in a controlled environment. There are a few limitations worth to be mentioned.

First, in the prototype, a local server was introduced between the gateway layer and the IOTA nodes. The aim was to handle the large amount of raw acceleration data and simplify the testing process. In practical application, this local server can be excluded. The sensor data can be published to the Tangle directly from IoT devices or through a gateway such as smartphone or Raspberry Pi. This could enable the real machine-to-machine communication and make it easier for large scale implementation.

Another limitation of this pilot study is that a public IOTA node was used for publishing and receiving data over the Tangle. The lagging time varied depending on the workload of that public node, which is always stable. In practical implementation, a private node should be set up according to the practical requirements.

### Conclusions

IOTA Tangle, together with the MAM communication protocol, could provide a fee-less, secure, highly scalable, and quantum-immune data sharing platform. The fast development of IoT is upgrading health care industry from digital to intelligent. The converging of IOTA Tangle, MAM, and IoT could significantly accelerate the health data sharing and pave way to realizing the vision of intelligent health care. The proposed solution in this study overcomes many of the challenges faced by other traditional block-based solutions in terms of cost, efficiency, scalability, and flexibility in data access management. It could be applied in many scenarios of health care, such as remote diagnosis, chronic disease monitoring, and elderly caring, as introduced in the previous ET diagnosis experiment. Patients can publish their own health data to the Tangle with different encryption options and authorize medical experts to access to the tremor and activity data during a period. Experts can also share the diagnosis result with patients or their relatives.

This solution could be useful in many other areas such as rehabilitation, sports and fitness, and labor health protection in workplaces, which indicates the directions for future work. For example, wearable devices can be used to monitor workers’ positions, activities, working load, and health indicators such as heart rate and blood pressure. Environmental sensors can be used to monitor the working conditions including the air quality, temperature, humidity, noise, and illumination. All these data or the periodical statistic results can be published to the Tangle and authorize access to different stakeholders such as Environmental Health and Safety experts, production managers, and government audit departments to better understand the health status of workers and avoid overfatigue or injuries.

## Abbreviations

DLT: distributed ledger technologies
ET: essential tremor
IoT: Internet of Things
MAM: Masked Authenticated Messaging
MHT: Merkle Hash Tree
OTS: one-time signature
